# Peripheral Nerve Blockade for Trimalleolar Ankle Fracture Surgery in a Third-Trimester Pregnancy: A Case Report

**DOI:** 10.7759/cureus.103626

**Published:** 2026-02-14

**Authors:** Ana M Milosavljevic, Milena Jovic, Andreja Baljozovic

**Affiliations:** 1 Anesthesiology and Critical Care, Institute for Orthopedics Banjica, Belgrade, SRB; 2 Orthopedics and Traumatology, Institute for Orthopedics Banjica, Belgrade, SRB

**Keywords:** anesthesia, ankle fracture, nonobstetric surgery, peripheral nerve block, pregnancy

## Abstract

Nonobstetric surgery of pregnant patients is a challenge and concern for nonobstetric anesthesiologists. The approach to the pregnant patient for nonobstetric surgery must be multidisciplinary, including an obstetrician, an anesthesiologist, and a surgeon. We present the case of a 21-year-old female patient, in the 37th week of pregnancy, who sustained a trimalleolar fracture of the ankle joint, which required surgical treatment. The operation was performed under peripheral nerve blockade with moderate sedation. For the popliteal sciatic block, 15 mL of 0.5% levobupivacaine and 10 mL of 1.3% lidocaine were administered. For the saphenous block, 10 ml of 0.5% levobupivacaine was given. Sedation was performed with intravenous boluses of propofol. Pre- and postoperative assessments included referral to the gynecology clinic, where fetal heart rate monitoring and uterine contractions were monitored. Postoperative pain was managed with 1 g of paracetamol intravenously every eight hours, and a single dose of 50 mg tramadol intravenously was given, eight hours after surgery, when the numeric rating scale (NRS) was 4. The primary aim was to ensure the safety of the mother and fetus. Peripheral nerve blockade reduces the neuroendocrine response to stress, reducing the need for opioids and systemic analgesics. Also, the risk of pulmonary aspiration, hypotension, and hypoxia is avoided. Well-planned anesthesia and postoperative analgesia are essential to protect the mother and fetus.

## Introduction

Orthopedic trauma during pregnancy has a low incidence (1-6%). Most often, trauma occurs in the third trimester. However, trauma increases the risk of complications, such as premature birth, placental abruption, uterine rupture, and fetal death. The leading cause of trauma during pregnancy is traffic accidents, violence, and falls [1,2].

Nonobstetric surgery of pregnant patients is a challenge and concern for nonobstetric anesthesiologists. The main goal is to ensure the safety of the mother and fetus. It is essential to avoid dangerous drugs, hypoxia, and hypotension while maintaining adequate uteroplacental perfusion. Regional anesthesia may be a better choice than general anesthesia because it reduces fetal exposure to drugs, avoids the risk of pulmonary aspiration, and provides better postoperative pain control. Regional anesthesia, including neuraxial (spinal and epidural) anesthesia and peripheral nerve blocks (PNBs), reduces the neuroendocrine response to stress and the need for opioids and systemic analgesics. PNB has a lesser influence on hemodynamics, thereby maintaining adequate uteroplacental perfusion [3].

To date, only a few papers have been written about ankle fracture surgery in the third trimester of pregnancy, with literature data on anesthesia methods being very scarce [4-7]. In this paper, we present the anesthesia management for ankle fracture surgery in a pregnant patient in the last trimester with a detailed description of the PNBs. This article was previously presented as a poster at the 41st Annual Congress of the European Society of Regional Anaesthesia and Pain Therapy (ESRA) in Prague, Czech Republic, 2024.

## Case presentation

**Figure 1:**
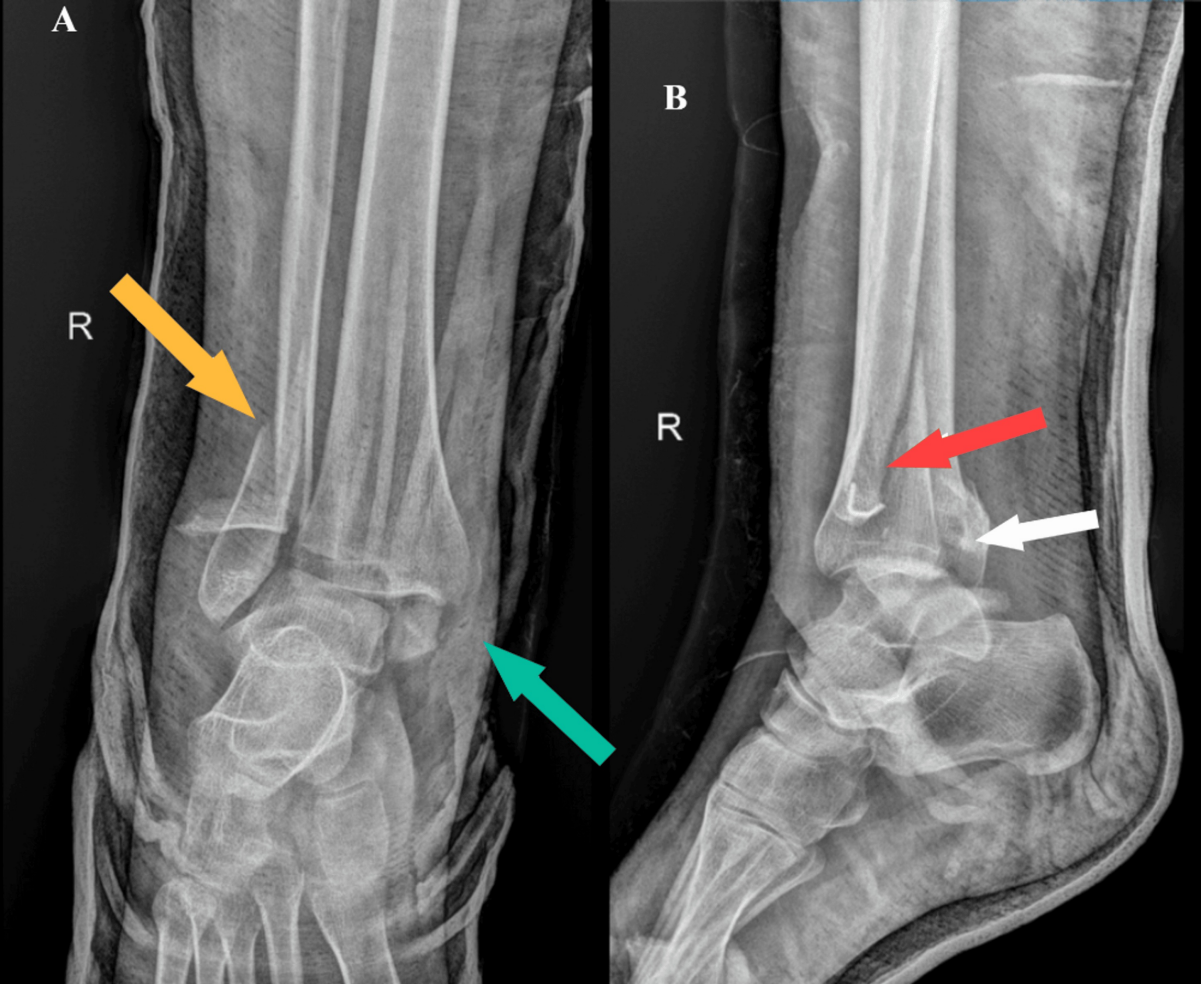
A radiography of the right ankle in anteroposterior (A) and lateral (B) views before surgery The arrows in the picture show the fracture sites of the distal tibia and fibula. In picture A (anteroposterior view), the yellow arrow indicates a fracture of the fibula-lateral malleolus. The blue arrow shows a fracture of the tibia, the medial malleolus. In picture B (lateral view), the red arrow shows a fracture of the lateral malleolus. The white arrow indicates a fracture of the tibia, the posterior malleolus

A 21-year-old female patient, in the 37th week of gestation, was admitted due to pain in her right ankle and inability to walk, after falling on the same level on the same day. The patient's symptoms included pain, swelling, deformity, and inability to bear weight on the injured leg. A radiograph of the right ankle joint showed a trimalleolar fracture with possible syndesmosis disruption (Figure 1). This type of fracture is a serious ankle joint injury, which includes a fracture of the fibula, lateral malleolus and a fracture of the tibia, as well as the medial and posterior malleolus. As this was an unstable fracture, it required surgical treatment.

Written informed consent was obtained. First, the patient was referred to the gynecology clinic, where fetal heart rate (FHR) and uterine contraction monitoring were performed before the procedure. The obstetrician assessed the fetus in good condition and the absence of uterine contractions. Then, screening for venous thromboembolism was performed due to the increased risk of deep vein thrombosis during pregnancy. After an anesthesiologist assessment, the patient was classified as American Society of Anesthesiologists (ASA) 2, according to the latest 2026 ASA guidelines [8]. Crystalloid infusions, proton pump inhibitors (pantoprazole 40 mg by intravenous injection), and an antibiotic (first-generation cephalosporin, cefazolin, 50 mg/kg/day intravenously) were started. The patients underwent a fracture surgery within the first three hours after injury. Standard noninvasive monitoring was conducted (noninvasive blood pressure, continuous electrocardiogram, and pulse oximetry). Patient characteristics and clinical data are summarized in Table 1.

**Table 1:** Anesthesia and surgery characteristics

Parameter	Data
Local anesthetic for popliteal sciatic nerve block	Levobupivacaine; lidocaine
Concentration of levobupivacaine for popliteal sciatic nerve block	0.5%
Volume of levobupivacaine for popliteal sciatic nerve block	15 ml
Dose of levobupivacaine for popliteal sciatic nerve block	75 mg
Concentration of lidocaine	1.3%
Volume of lidocaine	10 ml
Dose of lidocaine	13 mg
Local anesthetic for saphenous nerve block	Levobupivacaine
Concentration of levobupivacaine for saphenous nerve block	0.5%
Volume of levobupivacaine for saphenous nerve block	10 ml
Dose of levobupivacaine for saphenous nerve block	50 mg
Block coverage	L4-S2
Anesthesia type	Regional anesthesia
Type of surgery	Open reduction and internal fixation with locking plate
Surgery duration	60

The anesthesia plan included a femoral and popliteal nerve block with moderate sedation with propofol. To avoid potential hypotension from aortocaval compression by the pregnant uterus, the patient was positioned in left lateral decubitus, with the left side down. An ultrasound-guided popliteal sciatic block (eZono®4000; eZono AG, Jena, Germany) targeting its branches, the tibial nerve and the common peroneal nerve, was performed. Levobupivacaine 0.5% 15 ml (75 mg) and lidocaine 1.3% 10 ml (13 mg) were injected. Then, a proximal saphenous nerve block, which is a terminal sensory branch of the femoral nerve, was performed with levobupivacaine 0.5% 10 ml (50 mg), because blockade of the saphenous nerve achieves anesthesia for the cutaneous medial leg and ankle joint capsule.

**Figure 2:**
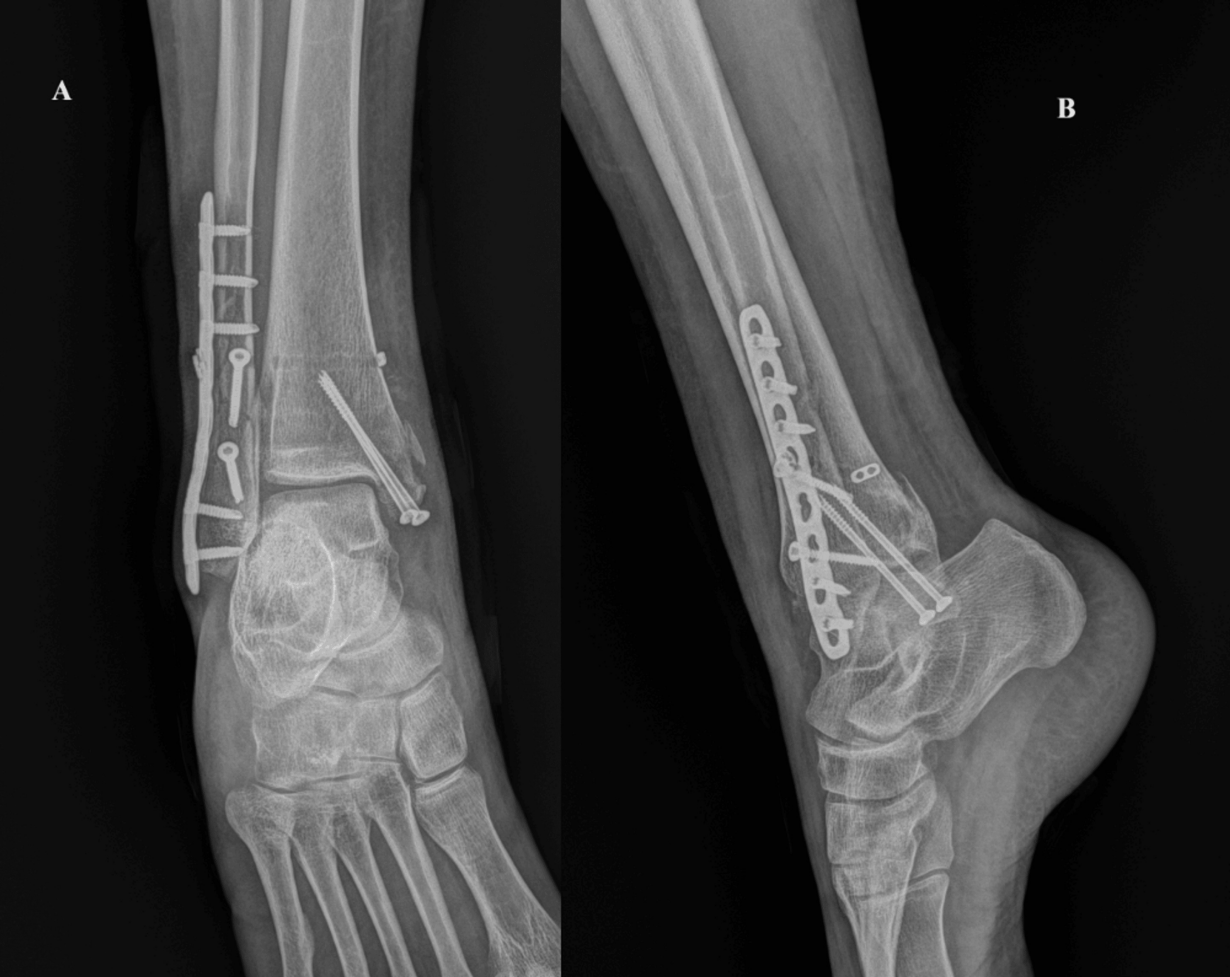
A radiography of the right ankle in anteroposterior (A) and lateral (B) views after fracture surgery This image shows fixed fractures of the malleolus with plate and screws

During the operation, the patient was sedated with intravenous propofol boluses. An initial propofol dose of 40 mg was given, followed by 10-20 mg boluses as needed. Spontaneous breathing was maintained, oxygen at 6 L/min was delivered by face mask, and intravascular volume was supported with Ringer’s lactate solution. The patient was positioned in the left lateral decubitus position, with a splint under the right hip and the table tilted left. A tourniquet was not used during the surgery. Ankle injury was treated with open reduction and internal fixation of the lateral and medial malleolus with a locking plate and two lag screws, as well as two cannulated cancellous screws, respectively. Tibiofibular syndesmosis was reduced and fixated with the Syndesmosis TightRope implant system (Arthrex Inc., Naples, FL). Posterior malleolus involved less than 25% of the articular surface and did not require fixation. Postoperative radiographs are shown in Figure *2*. Surgery duration was 60 minutes.

Postoperatively, the patient was observed in the postanesthesia care unit. Pain intensity was assessed using a numeric rating scale (NRS) [9]. After surgery, the NRS was 0. Three hours after surgery, the pain was described as 2 on the NRS. The analgesia plan included paracetamol 1 g intravenously, every eight hours; a single shot of tramadol 50 mg intravenously was given, eight hours after surgery, when the NRS was 4. Nonsteroidal anti-inflammatory drugs were not given due to the risk of premature closure of the ductus arteriosus in late pregnancy. Two hours after the operation, the patient was sent to the gynecology clinic, where FHR and uterine contraction monitoring were performed again, and after the procedure, the good condition of the fetus and the absence of uterine contractions were also assessed. The day after the operation, she was discharged to home treatment, with daily FHR monitoring. Thromboembolic prophylaxis with nadroparin calcium (3800 IU/0.4 ml) once daily was prescribed for 21 days. At 41 weeks of gestation, the patient vaginally delivered a healthy male infant.

## Discussion

Studies estimate that there are around 2.9 maternal fractures/10,000 live births, and most of them are classified as minimal trauma fractures during the second and third trimester. Lower limb is affected in 60% of cases, with predominance of ankle fractures in 39%, being the most common fracture location overall in pregnant women. More than half of all fractures that occurred in the third trimester require surgery [10]. According to a study by Dunning et al., 27% of pregnant women will experience a fall during pregnancy. This highlights a risk to the safety and well-being of both mother and child [11].

In addition to numerous physiological changes, joint biomechanics also change during pregnancy. The ligaments become more elastic, and walking kinematics and balance are influenced by pregnant morphology, with these changes especially apparent in the third trimester [12]. There are also alterations in sitting and standing posture, as well as during gait. Hip, knee, and ankle movements are altered in all planes, as well as torso and pelvic relationships [1,13]. Pregnant women show an increased mediolateral shift of the center of posture and reduced gait velocity, which explains the increased falling trend [13].

Literature data regarding anesthesia protocols in the third trimester are insufficient and rely only on a small number of case reports. Uzunov et al. primarily focus on surgical aspects, noting only that the operation was performed under spinal anesthesia [4]. One of the latest articles from Kasai et al. also describes ankle surgery performed under a combination of spinal and epidural anesthesia [5]. Güngör et al. focus on the anesthetic management for surgery to treat a pseudoaneurysm of the plantar media artery of the right foot. A popliteal sciatic nerve block was utilized during the operation with levobupivacaine 0.375% 30 ml, achieving a good sensory and motor block [6]. Finally, the article by Schwarzkopf et al. shows a patient operated on for an ankle fracture at 36th weeks of pregnancy, performed under spinal anesthesia, which was complicated by fetal distress, which required an emergency caesarean section [7].

Caring for a traumatized pregnant woman requires a multidisciplinary approach: an obstetrician, an anesthesiologist, and an orthopedic surgeon. The anesthesiologist must preserve the maternal physiology to maintain stable vital parameters. Next, it is necessary to maintain adequate uteroplacental perfusion in order to avoid fetal asphyxia. Furthermore, the selection of drugs must be careful and adapted to the trimester of pregnancy. It is necessary to avoid teratogenic drugs and drugs that can harm the fetus. The pregnant woman should be placed in the left lateral position to avoid aortocaval compression. It is necessary to improve maternal oxygenation and avoid hypotension [14,15]. The anesthesia will be chosen depending on the general condition of the pregnant woman, the type, and place of the surgical procedure. Regional anesthesia is always a better choice, because in this way, endotracheal intubation, a potentially difficult airway, and the risk of pulmonary aspiration are avoided, and less exposure of the fetus to anesthetic agents [15]. Adequate conditions for ankle fracture surgery could be achieved with regional anesthesia, which can be performed with a neuraxial block or a PNB. Both types of regional anesthesia provide conditions that reduce the neuroendocrine response to stress, with adequate pain control that is achieved both perioperatively and postoperatively, which reduces the need for systemic analgesics [15,16]. PNB has a lesser impact on vital organs, and hemodynamic stability is preserved. It is of great importance, considering that hypotension leads to reduced uteroplacental perfusion [14]. This type of anesthesia preserved the hemodynamic stability of the mother and the fetus with the absence of maternal hypotension, which was expressed during the introduction of spinal and general anesthesia.

The pharmacokinetics and pharmacodynamics of drugs change during pregnancy. Due to hemodilution, the plasma albumin concentration decreases, leading to increased free drug concentrations. Due to increased sensitivity to local anesthetics, it is necessary to reduce the usual dose to prevent toxicity in the supratentorial region. Local anesthetics easily cross the placenta and reach the blood of the fetus. Due to the lack of glycoprotein in the fetus, the fraction of free local anesthetics is increased, which can cause bradycardia [6,15]. Only the free, unbound fraction of the anesthetic can pass through the placenta. An amide type of local anesthetics that we use, bupivacaine and levobupivacaine, crosses the placenta more poorly than lidocaine. Common doses of local anesthetics have been shown to be teratogenic in female rats, but no effects on the fetus have been demonstrated in healthy pregnant women. Local anesthetics can be used in pregnancy, but only carefully, preferably at minimum effective doses [17-19]. According to that, we did not exceed the maximal levobupivacaine dosage (2 mg/kg) and lidocaine dosage (4 mg/kg).

We measured pain intensity using the NRS. Our analgesia plan included paracetamol and tramadol. In pregnancy, the drug of choice is paracetamol. In the last trimester of pregnancy, the use of NSAIDs should be avoided, as they can lead to premature closure of the ductus arteriosus, which can have a fatal outcome. Opioid analgesics can be used short-term, in small doses, during pregnancy. No opioids' teratogenic and mutagenic effects have been described in humans [1,20].

It is true that a smaller amount of local anesthetic is required for performing spinal anesthesia than for peripheral nerve blockade. During preoperative preparation, the obstetrician advised avoiding maternal hypotension to preserve uteroplacental perfusion. PNB preserves hemodynamic stability in conjunction with spinal anesthesia. Orthopedic injuries and surgery are very painful; there is a need for analgesic therapy. PNB with levobupivacaine achieves long-lasting analgesia up to eight hours, thereby reducing the need for systemic analgesics; however, with spinal anesthesia, the analgesic effect lasts up to three hours. Two hours after the operation, the patient was again transported to the gynecology clinic for postoperative FHR and uterine contraction monitoring. Bearing in mind that it was necessary to maintain hemodynamic stability, avoid NSAIDs in pain therapy, and the need to transport the patient to the gynecological clinic, PNB seemed to be the best choice of anesthesia technique. As the ankle injury and surgery occurred on the same day, the patient was anxious and fearful for her health and the health of the fetus. Perioperative sedation was performed with small doses of propofol, because it is short-acting and rapidly cleared from the circulation.

The limitations of this study include the lack of pregnancy data and long-term follow-up, as well as the presentation of only one case. The surgery in conditions of peripheral nerve blockade of our patient was without complications for the mother and the fetus, but this fact cannot be generalized to other pregnant women based on one case.

## Conclusions

The task of the anesthesiologist is to provide adequate conditions for the operation, while ensuring the safety of the mother and the fetus. For ankle fracture surgery, popliteal sciatic and saphenous nerve blocks were sufficient, with the addition of propofol sedation. This combination created favorable conditions for surgery, maintained the mother's hemodynamic stability, provided effective postoperative analgesia and early mobilization, and did not affect the fetus. A satisfactory overall outcome was achieved through a careful, multidisciplinary approach.
